# Tacrolimus reverses pemphigus vulgaris serum-induced depletion of desmoglein in HaCaT cells via inhibition of heat shock protein 27 phosphorylation

**DOI:** 10.1186/s12865-023-00582-z

**Published:** 2023-11-08

**Authors:** Zhimin Xie, Xiangnong Dai, Qingqing Li, Sifan Lin, Xingdong Ye

**Affiliations:** 1 Department of Dermatology, The Fifth Affiliated Hospital of Guangzhou Medical University, Guangzhou, Guangdong China; 2 Department of Dermatology, Institute of Dermatology, Guangzhou Medical University, Guangzhou, Guangdong China; 3 Department of Dermatology, Guangzhou Institute of Dermatology, Guangzhou, Guangdong China

**Keywords:** Pemphigus Vulgaris, Desmoglein, Tacrolimus, Acantholysis, Glucocorticoids

## Abstract

**Background:**

Glucocorticoids are the first-line treatment for Pemphigus vulgaris (PV), but its serious side effects can be life-threatening for PV patients. Tacrolimus (FK506) has been reported to have an adjuvant treatment effect against PV. However, the mechanism underlying the inhibitory effect of FK506 on PV-IgG-induced acantholysis is unclear.

**Objective:**

The objective of this study was to explore the effect of FK506 on desmoglein (Dsg) expression and cell adhesion in an immortalized human keratinocyte cell line (HaCaT cells) stimulated with PV sera.

**Methods:**

A cell culture model of PV was established by stimulating HaCaT cells with 5% PV sera with or without FK506 and clobetasol propionate (CP) treatment. The effects of PV sera on intercellular junctions and protein levels of p38 mitogen-activated protein kinase (p38MAPK), heat shock protein 27 (HSP27), and Dsg were assayed using western blot analysis, immunofluorescence staining, and a keratinocyte dissociation assay.

**Results:**

PV sera-induced downregulation of Dsg3 was observed in HaCaT cells and was blocked by FK506 and/or CP. Immunofluorescence staining revealed that linear deposits of Dsg3 on the surface of HaCaT cells in the PV sera group disappeared and were replaced by granular and agglomerated fluorescent particles on the cell surface; however, this effect was reversed by FK506 and/or CP treatment. Furthermore, cell dissociation assays showed that FK506 alone or in combination with CP increased cell adhesion in HaCaT cells and ameliorated loss of cell adhesion induced by PV sera. Additionally, FK506 noticeably decreased the PV serum-induced phosphorylation of HSP 27, but had no effect on p38MAPK phosphorylation.

**Conclusion:**

FK506 reverses PV-IgG induced-Dsg depletion and desmosomal dissociation in HaCaT cells, and this effect may be obtained by inhibiting HSP27 phosphorylation.

**Supplementary Information:**

The online version contains supplementary material available at 10.1186/s12865-023-00582-z.

## Introduction

Pemphigus is a group of rare, chronic, potentially life-threatening, autoimmune bullous diseases. The two main pemphigus variants are pemphigus vulgaris (PV), which often originates with painful oral erosions, and pemphigus foliaceus (PF), which is characterized by exclusive skin lesions [1]. IgG antibodies against epidermal desmosomes, including desmoglein (Dsg) 1 and Dsg3, two major cell-cell adhesive structure proteins, cause loss of intercellular adhesion, acantholysis, and blister formation [2, 3]. Furthermore, both anti-Dsg1 and -Dsg3 antibodies are involved in the activation of signaling pathways including Ca^2+^, p38 mitogen activated protein kinases (p38MAPK), protein kinase C (PKC), Src, EGFR/Erk, and several others, resulting in modulation of keratinocyte cell adhesion properties [4].

The MAPK family, a group of highly conserved protein kinases that play an important role in signal transduction by regulating gene transcription in the nucleus in response to changes in the cellular environment, includes four distinct cascades, namely, the extracellular signal-related kinases (ERK1/2), ERK5, p38MAPK, and Jun N-terminal kinases (JNK1/2/3) [5, 6]. p38MAPK inhibition blocks loss of cell cohesion in keratinocyte cultures and blister formation in mouse models induced by PV autoantibodies [7]. The pathogenesis of PV may be related to the activation of p38MAPK and subsequent phosphorylation of the heat shock protein 27 (HSP27), which leads to actin cytoskeleton reorganization and keratin retraction [8]. Specific inhibition of this molecule using SB203580, a selective and ATP-competitive p38 MAPK inhibitor, was effective in preventing loss of adhesive contacts in mouse models [9].

The annual incidence of PV ranges between 0.76 and 32.0 cases per million population, and the epidemiology of PV accounts for about 65–95% of pemphigus cases [10]. Traditionally, PV has been treated with a combination of high-dose systemic glucocorticoids and an adjuvant immunosuppressant. An anti-CD20 antibody, rituximab, is recommended as the first-line treatment for moderate-to-severe PV [11]. Because the long-term administration of glucocorticoids can cause adverse side effects, such as secondary infection, osteoporosis, and obesity, a more effective treatment for PV is needed.

Tacrolimus (FK506) is a calcineurin inhibitor that is used to suppress the immune system in a variety of conditions, including treatment of diverse immune disorders, inhibiting the formation of activated T cells and suppressing the synthesis of pro-inflammatory cytokines [12]. Previous studies have investigated the safety and effectiveness of systemic FK506 or topical use in pemphigus diseases [13–17]. In a study with PV model mice, FK506 monotherapy showed satisfactory effects by inhibiting anti-Dsg3 IgG production, the appearance of the PV phenotype, and development of body weight loss [18]. This study aimed to explore the effect of FK506 and the highly potent glucocorticoid—clobetasol propionate (CP)—on the intercellular junctions and Dsg3 expression in HaCaT cells stimulated with sera from PV patients.

## Results

### PV sera- and AK23-induced loss of keratinocyte adhesion and Dsg3 depletion

We initially characterized changes in Dsg3 expression in HaCaT cells incubated with PV sera over a time course. AK23 is a monoclonal antibody (mAb) obtained by immunizing mice with recombinant mouse Dsg3 ectodomain and is currently widely used to model the pathogenesis of PV [19]. In this study, AK23 was used as a positive control.

**Fig. 1:**
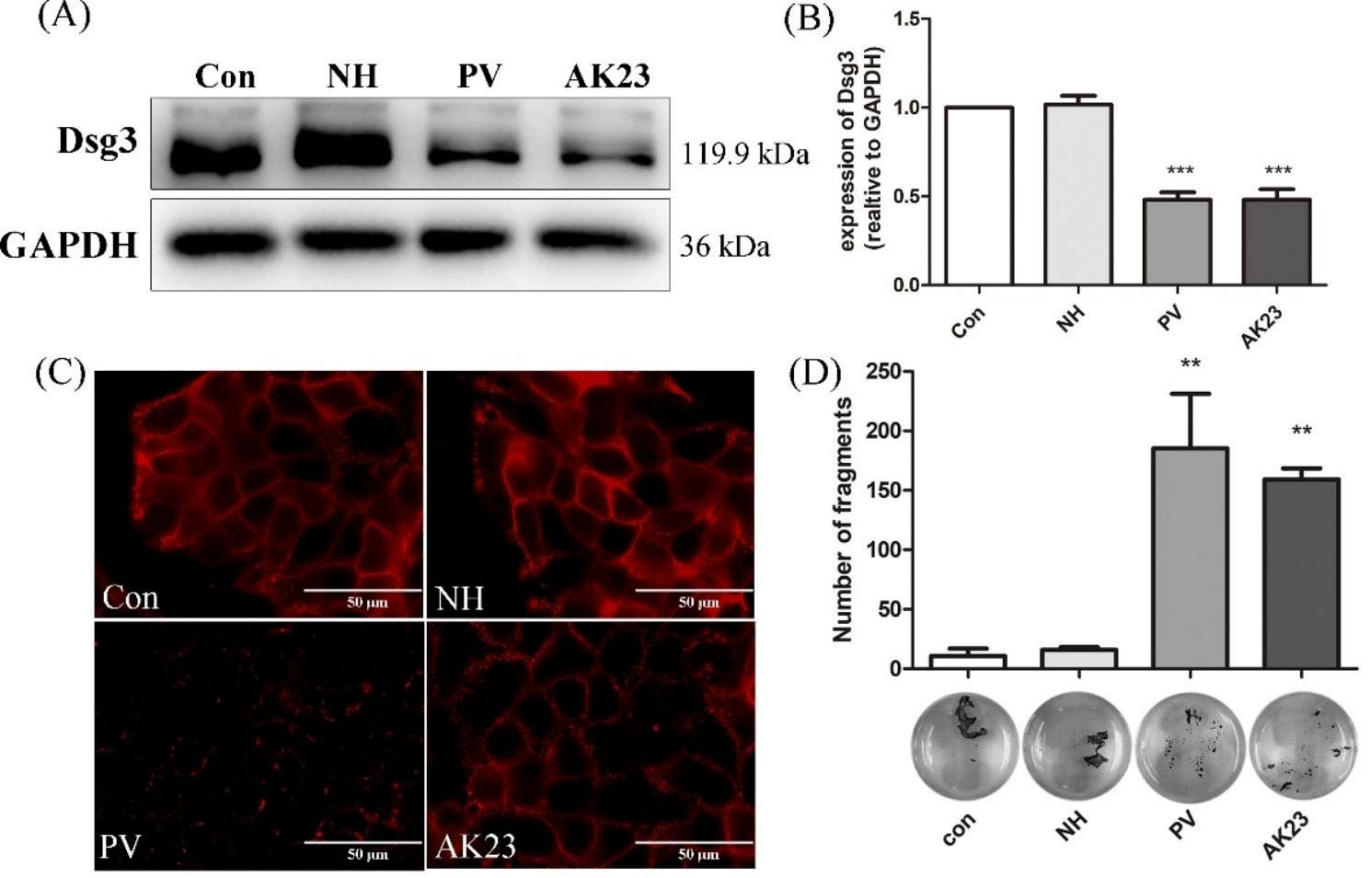
Both pemphigus sera and AK23 caused a decrease in keratinocyte adhesion and Desmoglein (Dsg) 3 internalization in HaCaT cells. (**A**) Western blot analysis of Dsg3 in total cell lysates of HaCaT cells incubated with 5% PV sera or 1 µg/ml AK23 for 24 h. GAPDH was used as a loading control. Respective controls [normal control group (Con) or normal healthy sera group (NH)] were run in parallel. Both PV sera and AK23 decreased Dsg3 protein levels as compared with the control. (**B**) Densitometric analysis was derived from the ratio of Dsg3 to GAPDH expression in each group and then normalized to the control group. Statistically significant differences are indicated by *** p < 0.001 vs. Con. Data are represented by mean ± SE (n = 3). (**C**) Immunostaining revealed that a 24-h incubation with 5% PV sera or 1 µg/ml AK23 induced fragmentation of the Dsg3 staining pattern along the cell borders (scale bar = 50 μm). (**D**) Dissociation assays in HaCaT cells showed weaker intercellular adhesion consistence with more free cells in cells incubated with 5% PV sera or AK23. ** p < 0.01 vs. Con. Data are shown as mean ± SE (n = 3)

The protein expression of Dsg3 was dramatically decreased after the addition of PV sera at 1 h, and Dsg3 protein levels remained low at 24 h (Supplementary Fig. 1). Dsg3 expression in HaCaT cells was significantly decreased in the PV group and AK23 group when compared with the control group and normal healthy (NH) group (Fig. 1A–B). Fluorescent monoclonal antibody detection showed that Dsg3 was continuously distributed along cellular junctions under control conditions or following treatment with healthy donor sera. In contrast, 5% PV sera or 1 µg/mL AK23 treatment resulted in the disruption of Dsg3 protein expression, the linear fluorescence of Dsg3 on the surface of cells disappeared, and the fluorescent particles were aggregated on the cell surface and cytoplasm membrane (Fig. 1C). However, AK23 showed a weaker effect on the adhesion of HaCaT cells as compared to PV sera.

Cell-cell adhesion was also compromised as revealed by the results of the dispase-based dissociation assays. In agreement with the change in Dsg3 expression, cell adhesion was significantly impaired upon the addition of PV sera and AK23 (Fig. 1D). PV sera resulted in a slight increase in the number of fragments in HaCaT cells compared to AK23, however, the comparison was not statistically significant. Taken together, these results demonstrated that HaCaT cells manifest the typical hallmarks of PV when exposed to PV sera or AK23.

**Fig. 2:**
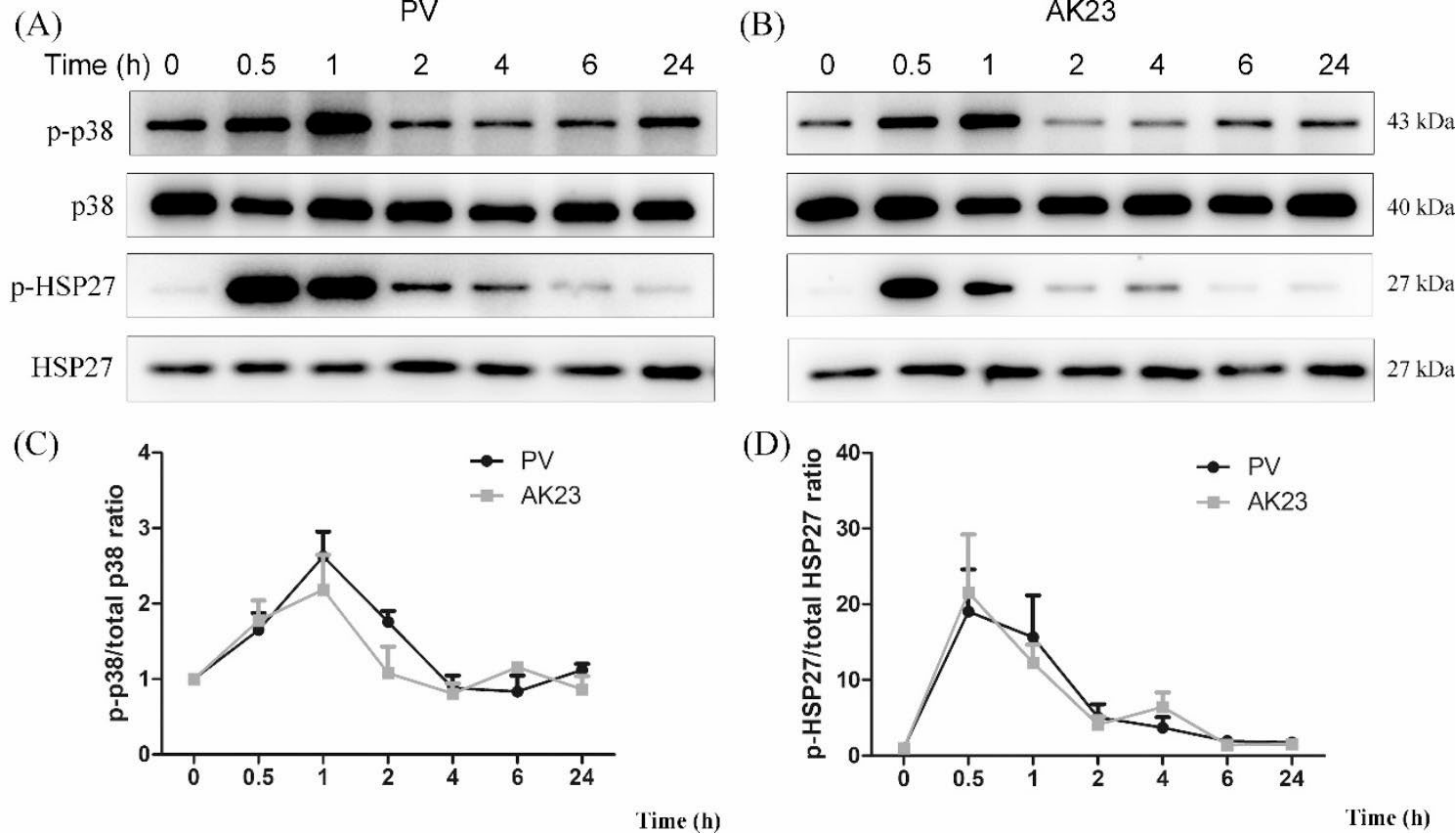
Time course of p38 mitogen-activated protein kinase (MAPK) and heat shock protein (HSP) 27 activation in HaCaT cells treated with pemphigus vulgaris (PV) sera and AK23 monoclonal antibody. (**A, B**) (with the exception of 0 h group as blank control ant it is not treated with any reagent), Peaks of phospho-p38 MAPK (p-p38 MAPK) and phospho-HSP27 were detected by western blot. (**C, D**) Densitometric analysis was derived from the ratios of p-p38 MAPK to p38 MAPK and p-HSP27 to HSP27 expression in each group and normalized to the 0 h group. Peak activation of p38 MAPK and HSP27 by PV sera and AK23 occurred around 0.5–2 h. Levels of total p38 MAPK and HSP27 are shown as loading controls (n = 3)

In order to explore which proteins are involved in the pathogenesis of PV, we measured the phosphorylation levels of p38 MAPK and HSP27 in HaCaT cells, using total p38MAPK and total HSP27 as a loading control, respectively. Both PV serum and AK23 induced rapid phosphorylation of p38 MAPK and HSP27, with peak activation at 0.5–2 h (Fig. 2).

### FK506 inhibited PV sera- and AK23 induced-disruption of cell-cell contact and Dsg3 internalization

**Fig. 3:**
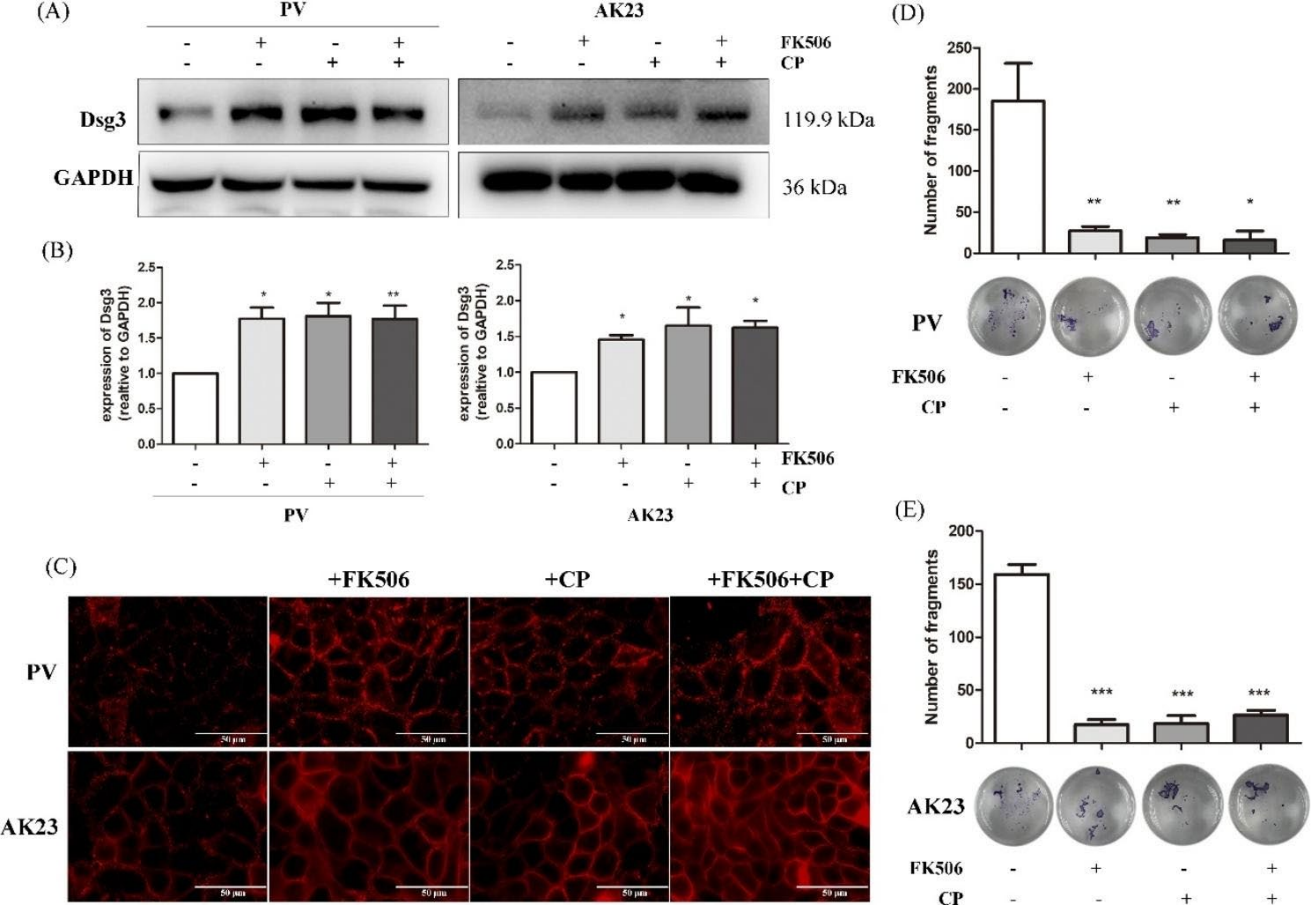
FK506 and clobetasol propionate (CP) rescue disruption of cell-cell contacts and the loss of desmoglein (Dsg) 3 induced by pemphigus vulgaris (PV) sera and AK23. HaCaT cells were co-incubated with 100 nM FK506 and/or 1 µM CP in the present of 5% PV sera or 1 µg/mL AK23 for 24 h prior to western blot and immunostaining. (**A**) Both PV sera- and AK23-stimulated Dsg3 internalization was blocked by FK506 and CP. (**B**) Statistically significant differences are indicated by * *p* < 0.05 and * *p < 0.01 vs. PV group or AK23 group, respectively. Data are represented by mean ± SE. (**C**) Consistent with western blot, FK506 and/or CP prevented the PV sera- and AK23-mediated loss of cell surface-localized Dsg3. (scale bar = 50 μm). (**D** and **E**) Dispase-based dissociation assays indicated that when cells in the PV group and AK23 group were treated with FK506 and/or CP, the number of free cells was dramatically decreased (n = 3). Statistically significant differences are indicated by *** *p* < 0.001 vs. PV group or AK23 group, respectively

The results of the western blot demonstrated that expression of Dsg3 was dramatically increased when HaCaT cells stimulated with PV sera or AK23 were treated with FK506 and/or CP (Fig. 3A and B). Consistent with these findings, FK506 and/or CP prevented the PV sera-mediated loss of cell surface-localized Dsg3 (Fig. 3C). In the AK23 group, the addition of FK506 and/or CP restored the linear fluorescent distribution of Dsg3 (Fig. 3C). Dispase-based dissociation assays indicated that when cells in the PV sera and AK23 groups were treated with FK506 and/or CP, the number of free cells dramatically decreased (p < 0.05) (Fig. 3D–E).

### FK506 did not decrease the phosphorylation of p38 MAPK but inhibited the phosphorylation of HSP27 induced by PV sera

**Fig. 4:**
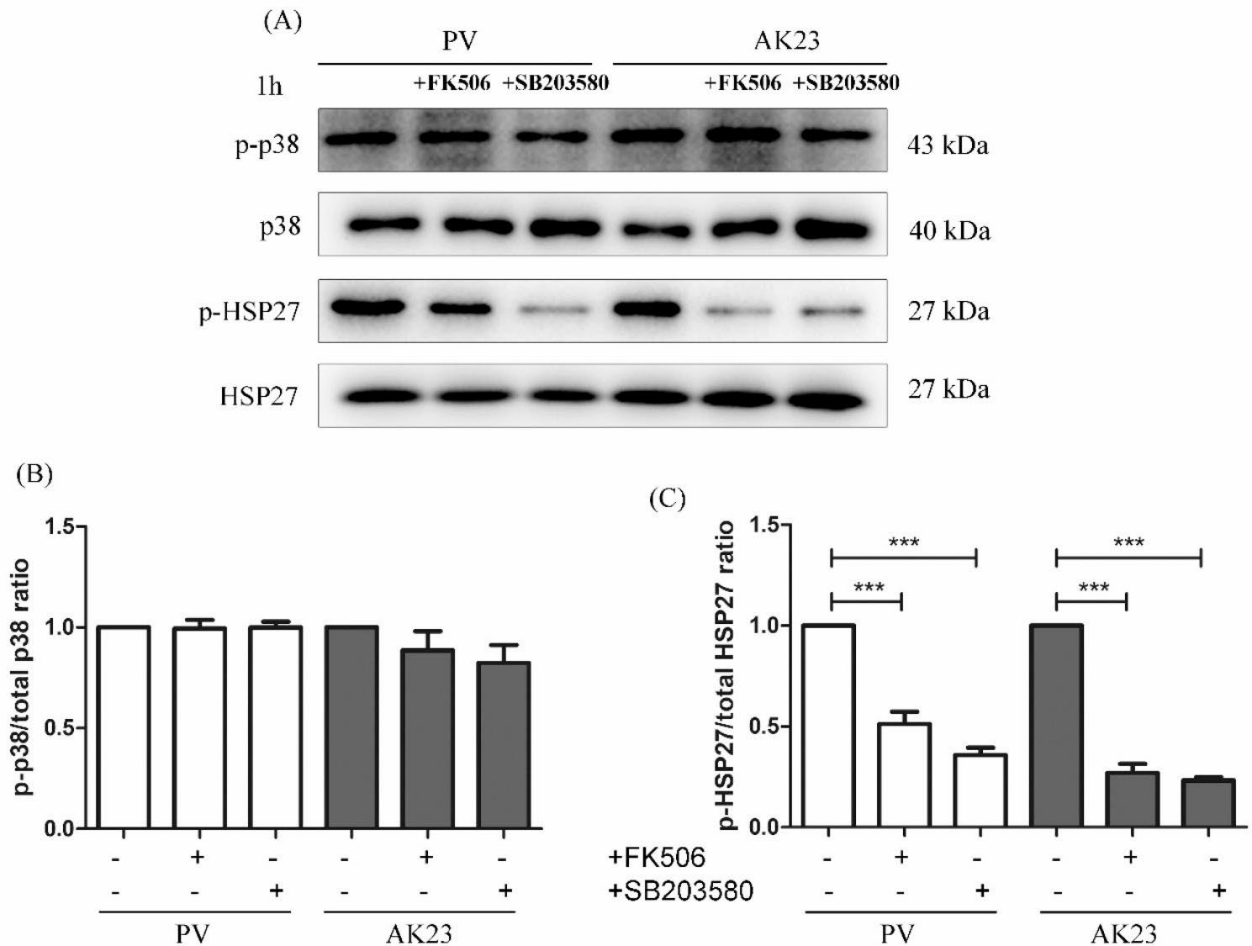
The effect of FK506 on the pemphigus vulgaris (PV) sera-induced phosphorylation of both p38 mitogen-activated protein kinases (MAPK) and heat shock protein (HSP) 27. HaCaT cells were pretreated with 100 nM FK506 or 1 µM SB203580 (p38 MAPK inhibitor) for 2 h, followed by exposure to 5% PV sera or 1 µg/ml AK23 for 1 h. FK506 significantly blocked the phosphorylation of HSP27, but not p38 MAPK. Levels of total p38 MAPK are shown as loading controls (n = 3). (**B** and **C**) Data are represented by mean ± SE.

We further analyzed the effect of FK506 on the phosphorylation of both p38 MAPK and HSP27 induced by PV sera. Pretreatment of HaCaT cells with 100 nM FK506 for 2 h, followed by exposure to PV sera or AK23 for 1 h. FK506 significantly blocked phosphorylation of HSP27, but not p38 MAPK (Fig. 4). These results suggested that FK506 may inhibit Dsg3 depletion through inactivation of HSP27.

## Discussion

**Fig. 5:**
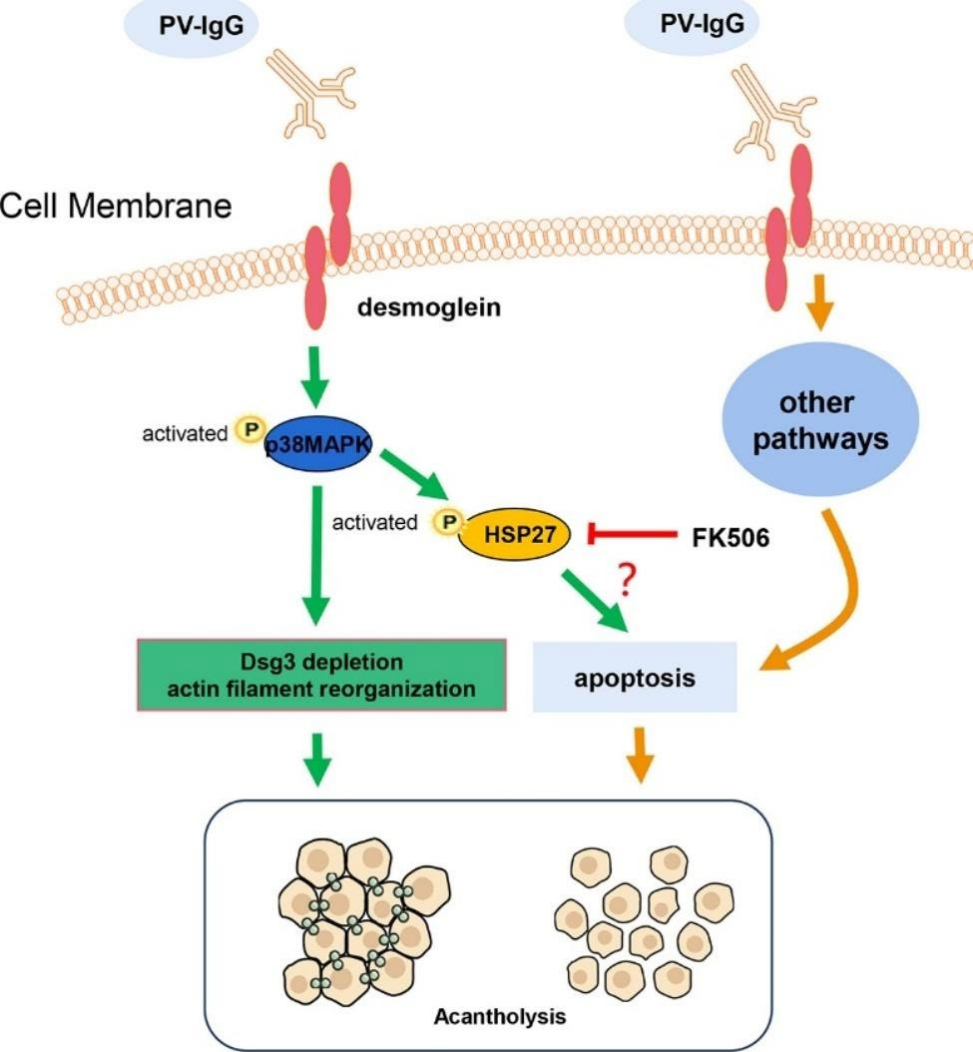
Pictorial presentation of the proposed hypothesis pertaining to apoptosis in the pathogenesis of pemphigus vulgaris. PV-IgG depletes desmoglein (Dsg)3 and causes actin filament reorganization, leading to acantholysis. FK506 blocks heat shock protein (HSP) 27 phosphorylation and prevents changes in Dsg3 depletion and actin filament reorganization

The objective of the present work was to investigate the effect of FK506 on the p38 MAPK and HSP27 pathways in an in vitro model of pemphigus [20]. FK506 inhibited pemphigus sera-induced loss of keratinocyte adhesion *in vitro.* We also found that PV serum and AK23 activated p38 MAPK and HSP27, producing a peak at 0.5–1 h. Activation of p38 MAPK and subsequent phosphorylation of HSP27 seem to be related to the pathogenesis of PV, as both lead to keratin retraction and reorganization of the actin cytoskeleton [21]. Though a protective effect was observed for FK506 on PV sera-induced acantholysis, our results herein showed that the phosphorylation of p38 MAPK was not inhibited by treatment with FK506. Our results indicate that FK506 inhibits acantholysis through a mechanism independent of p38 MAPK phosphorylation (Fig. 5).

FK506 inhibits calcineurin and the formation of activated T cells, suppresses the synthesis of pro-inflammatory cytokine, and is also associated with the inhibition of nitric oxide synthase activation, cell degranulation, and apoptosis [12, 22] and is widely used as an effective agent to treat autoimmune diseases [23, 24]. Studies have found beneficials results using systemic tacrolimus as an adjuvant treatment in pemphigus [16, 17]. FK506 was reported to have suitable tolerance as a steroid-sparing immunosuppressive agent in the management of PV [16, 17]. However, the mechanism underlying the effectiveness of FK506 for the treatment of PV needs to be further clarified. As far as we know, this is the first study assessing the effectiveness of FK506 for the treatment of pemphigus relating to the p38 MAPK pathway in an in vitro model of pemphigus.

The p38MAPK signaling pathway is an important signaling pathway mediating Dsg3 internalization followed by depletion by endosomes [25]. A previous study reported that the pathogenic activity of polyclonal PV IgG can be attributed to p38 MAPK-dependent clustering and Dsg3 endocytosis and that pathogenic monoclonal Dsg3 antibodies, such AK23, cause loss of adhesion primarily through hindrance, which do not function through p38MAPK pathway [26]. Activation of the p38 MAPK subsequently leads to keratin retraction and reorganization of the actin cytoskeleton, which appears to be involved PV pathogenesis [27]. As previously reported, PV sera induced acantholysis and decreased the total amount of Dsg3 in PV-IgG incubated keratinocytes [28]. Binding of autoantibodies to Dsg3 activated the p38MAPK pathway, resulting in Dsg3 depletion and a collapse in the keratin cytoskeleton [29, 30]. PV IgG causes the internalization of cell-surface Dsg3 into endosomes after binding to keratinocytes as early as 4 h post-exposure, and this phenomenon was able to be blocked by the p38 MAPK inhibitor, SB202190 [27, 30]. In addition, previous observations in tissue culture and animal models suggested a central role for p38 MAPK in the mechanisms underlying acantholysis in PV [29, 31, 32]. In the present study, PV sera and AK23 induced rapid phosphorylation of p38 MAPK, with peak activation at around 1 h (Fig. 2), which was consistent with previous studies [31, 33]. FK506 inhibits desmosomal dissociation of HaCaT cells, but it does not inhibit p38 MAPK phosphorylation pathway (Figs. 3 and 4). We postulated that the phosphorylation of HSP27 may promote the apoptosis pathway, which was inhibited by FK506, however, further studies are needed.

HSP27 belongs to the small molecular weight Hsp family, whose expression is induced in response to a wide variety of physiological and environmental insults [34]. The mammalian small Hsps are rapidly phosphorylated by MAPK-activated proteinase 2/3 (MAPKAP 2/3) at several distinct sites following exposure to a wide range of extracellular stresses [35]. HSP27 is involved in cell death pathways by regulating partner proteins, such as necrosis and apoptosis, and phosphorylated HSP27 regulates actin filaments dynamics and intracellular redox state in cytoskeleton organization during processes such as cell migration or cell stress [34]. A study recently reported a new pathological mechanism of acantholysis in pemphigus, i.e., apoptosis, which emphasizes the involvement of apoptotic enzymes in the occurrence of acantholysis in terms of molecular events and time series [36]. In the present study, the phosphorylation of HSP27 induced by PV sera was inhibited by FK506. However, immunofluorescence assays showed that silencing the *HSP27* gene (HSP27-HOMO-877, Gene ID: 3315) did not prevent Dsg3 endocytosis induced by PV sera on the surface of HaCaT cells (Supplementary Figs. 2 and 3). This suggests that HSP27 phosphorylation may be involved in HaCaT apoptosis but not directly involved in endocytosis of Dsg3, as inactivation of HSP27 is a less likely mechanism for acantholysis.

Recent studies have found that calcium (Ca^2+^) signaling is crucial to a variety of functions of biology. Ca^2+^ flux-dependent signaling pathways have been reported to play a vital role in the epidermal blistering, and inhibition of Ca^2+^ signaling blocked keratinocyte adhesion loss in vitro and human skin blistering ex vivo induced by pemphigus Dsg IgG. [2]. Though we are not sure whether FK506 inhibits calcium influx in cell membranes. FK506 has been attributed to have immunodulatory effects on both T cells and non-T cells, including keratinocytes [37]. FK506 may also reduce Dsg3 depletion through the inhibition of a Ca^2+^ pathway and deserves further investigation.

This study has some limitations. First, anti-Dsg1 antibodies were not used as another positive control. Second, although FK506 inhibits HSP27 phosphorylation and silencing the HSP27 gene does not prevent Dsg3 endocytosis, the question of whether FK506 affects HSP27 expression has not been answered. It remains unclear if FK506 inhibits desmosomal dissociation of keratinocytes through regulating phosphorylation of downstream targets, such as MAPKAP kinase 2/3.

## Conclusion

In summary, this study showed that PV sera disrupts desmosome junctions by inducing endocytosis in HaCaT cells, resulting in desmosomal dissociation. Furthermore, FK506 reversed this effect, which was comparable to CP. FK506-dependent inhibition of HSP27 phosphorylation may also contribute to enhanced cell-cell adhesion of the HaCaT monolayer. These findings lay the groundwork for elucidating the mechanism of action of FK506 in the treatment of pemphigus.

## Materials and methods

### Participants and specimens

Patient blood sera were collected from Guangzhou Institute of Dermatology between October 2019 and October 2020. All six patients were in the active stage prior to treatment, and blood sera from three healthy volunteers were used as healthy control. The characteristics and the titer of anti-Dsg1 and anti-D3 antibodies in the patient’s serum is shown in Table 1. The inclusion and exclusion criteria of PV patients have been reported in the literature [17, 38]. Disease severity was scored using the Pemphigus Disease Area Index (PDAI) score. The mixed serum was prepared by heating at 56 °C for 30 min to inactivate complement. An anti-Dsg antibody was tested by enzyme-linked immunosorbent assay (ELISA) assay. The anti-Dsg antibody concentration in the mixed serum was 169 U/mL for Dsg1 and 117 U/mL for Dsg3. Anti-Dsg antibody titers were negative in the mixed serum from the three healthy volunteers (Dsg1: 1 U/mL; Dsg3: 1 U/mL, 2 females and 1 males, 26–29 years old). This study and all relevant experiments were reviewed and approved by the Guangzhou Institute of Dermatology Research Ethics Committee (NO. 201,802, Guangzhou, Guangdong Province, China).

**Table 1:** Characteristics of the six PV patients included in the study

Factor	Characteristics
Gender	3 male and 3 female
Age (mean ± SD)	52.45 ± 15.20
anti-Dsg3 (U/ml, mean ± SD)	166.33 ± 52.69
anti-Dsg1(U/ml, mean ± SD)	20.22 ± 74.45
PDAI score (mean ± SD)	26.27 ± 8.10

### Antibodies and reagents

The primary antibodies included mouse anti-Dsg3 antibody (Abcam, Cambridge, UK), rabbit anti-p38 MAPK, rabbit anti-phospho-p38 MAPK (Thr180/Tyr182, Cell signaling Technology, Danvers, MA), mouse anti-HSP27, rabbit anti-phospho-HSP27 (Ser82, Cell signaling Technology), and rabbit anti-GAPDH (Affinity Biosciences, OH, USA). The secondary antibodies included a goat anti-mouse antibody, goat anti-rabbit antibody (Abclonal, Wuhan, China), and Cy3-conjugated goat anti-mouse (Abclonal) antibody.

Other regents included FK506 (Med Chem Express, Shanghai, China), clobetasol propionate (CP) (Macklin, Shanghai, China) and a p38 MAPK inhibitor, SB203580 (Med Chem Express). The pathogenic monoclonal antibody AK23 against Dsg3 was purchased (BML, Tokyo, Japan) and used at a concentration of 1 µg/ml as a positive control.

### Cell culture and study design

For all experiment, the human immortalized keratinocyte cell line HaCaT (MissBio Co., Ltd. Guangzhou, China) was used to establish an in vitro model of PV according to a previous study [39, 40]. Cells was maintained in Dulbecco’s modified Eagle’s medium (DMEM) supplemented with 5% fetal bovine serum (FBS) and 1% penicillin–streptomycin solution. HaCat cells were grown in a humidified atmosphere at 37 °C in a 5% CO2 incubator. After the cells reached confluence, the medium was changed, and cell monolayers were treated accordingly.

Four experimental groups were designated in the study: (i) normal control group (Con), which refers to the group that only received DMEM with 5% FBS medium; (ii) normal healthy sera group (NH), refers to the group with the presence of 5% healthy sera in DMEM medium; (iii) PV sera group (PV), refers to the group with the presence of 5% PV sera in DMEM medium; and (iv) AK23 group, refers to 1 µg/mL AK23 in 5% FBS DMEM medium. The HaCaT cells were sub-cultured until reaching 60–70% confluency, and exposed to different conditions as mentioned above for another 24 h. For FK506 studies, cells were incubated in 5% PV sera or AK23 with 100 nM FK506 and/or 1 µM CP.

### Dispase-based keratinocytes dissociation assay

The dispase-based keratinocyte dissociation assay is currently the main tool for the analysis of antibody-induced acantholysis in in vitro models of PV [41]. This assay was performed as previously described [2, 42, 43]. Briefly, HaCaT cells were seeded in 24-well plates and grown to 60–70% confluency and exposed to different conditions as mentioned above. After removing the medium and washing with pre-warmed Hank’s Balanced Salt Solution (HBSS, Solarbio, Beijing, China), 2.4 U/ml Dispase II (Solarbio, Beijing, China) in HBSS was incubated for 30 min at 37 °C, and cell monolayers were subsequently released from the well bottom. Each condition was treated for 30 min with thiazolyl blue tetrazolium bromide (namely, methylthiazol tetrazolium, MTT) (Sigma-Aldrich, Taufkirchen, Germany) at a final concentration of 10 µM to better visualize the monolayer sheets. After that, cell monolayers were exposed to mechanical stress by pipetting the monolayers 10 times with a 1-mL electric pipette (Eppendorf, Wesselingberzdorf, Germany) for the production of cell fragments. In each well, the resulting fragments were counted using a binocular microscope (CKX41, Olympus, Tokyo Japan). Each experiment was conducted three times.

### Western blotting assay

HaCaT cells were grown in 6-well plates for 18–24 h until the cultures reached 70–80% confluency, and then the medium was replaced with 5% FBS medium for the Con group, 5% healthy sera in DMEM medium for the NH group, 5% PV sera in DMEM medium for the PV sera group, and 1 µg/mL AK23 in 5% FBS DMEM medium for the AK23 group. For FK506 studies, cells were incubated in 5% PV sera or AK23 with 100 nM FK506 and/or 1 µM CP. After which, total cell lysates were prepared from HaCaT cells by extracting the proteins in RIPA buffer (Beyotime, Shanghai, China) with protease and phosphatase inhibitors. Proteins (20 µg) were separated by SDS-polyacrylamide gel electrophoresis (SDS-PAGE) and transferred to polyvinylidene fluoride (PVDF) membranes (Merck Millipore, Darmstadt, Germany). The PVDF membranes were blocked for 2 h with 5% non-fat milk in 0.1% Tris-buffered saline with Tween (TBST) and immunoblotted with primary antibodies (GAPDH 1:10000, Dsg3 1:1000, p38MAPK 1:1000, p-p38MAPK 1:1000, HSP27 1:1000, and p-HSP27 1:1000) at 4 °C for another 12–16 h. For phosphoproteins, the PVDF membranes were blocked with 5% bovine serum albumin (BSA) in 0.1% TBST.

### Immunofluorescence assay

The cells were grown to confluence on glass coverslips for 18–24 h in a 24-well plate. After 24 h of incubation with different culture medium as described above, the cells were washed with PBS and subsequently fixed in a 4% paraformaldehyde solution for 15 min then permeabilized with 0.1% Triton X-100 (in PBS) for 10 min. To prevent unspecific binding, a solution of 5% BSA was applied for 1 h at 25 °C before incubation with primary antibody (anti-Dsg3 antibody, 1:250 dilution) at 4 °C for 12–16 h. After three PBS washing steps, a Cy3-conjugated goat anti-mouse antibody was added as the secondary antibody (1:500 dilution) were applied for 1 h at 25 °C. A fluorescence microscope (BX63, Olympus) was used for image acquisition.

### Statistical analysis

Western blots were visualized and analyzed using Image J (NIH, Maryland, USA, http:imagej.nih.gov/ij). Statistical analysis was performed using a one-way ANOVA followed by Student-Newman-Keuls correction using GraphPad Prism 5 (GraphPad Software, La Jolla, CA, USA) for comparison of multiple groups. A *p* value < 0.05 was considered statistically significant. Data are represented as the mean ± SE from three independent experiments.

### Electronic supplementary material

Below is the link to the electronic supplementary material.

Supplementary Material 1

Supplementary Material 2

## Data Availability

The datasets used and/or analyzed during the current study are available from the corresponding author upon reasonable request.

## Abbreviations

PV: Pemphigus vulgaris
Dsg: Desmogleins
AZA: Azathioprine
MMF: Mycophenolate mofetil
CP: Cyclophosphamide
NH: Normal healthy
PBS: Phosphate-buffered saline
MAPK: Mitogen-activated protein kinase
HSP: Heat shock protein
DAPI: 4′,6-diamidine-2′-phenylindole dihydrochloride
