# Supplementary material for: Tacrolimus reverses pemphigus vulgaris serum-induced depletion of desmoglein in HaCaT cells via inhibition of heat shock protein 27 phosphorylation
Source: BMC Immunol. 2023 Nov 8;24:43. doi: 10.1186/s12865-023-00582-z (PMC10634089; doi:10.1186/s12865-023-00582-z)
Supplement: Supplementary file 1 — Supplementary Material 1 [file 12865_2023_582_MOESM1_ESM.pdf]

Supplementary Material

Supplementary Figure 1. After reaching 60–70% confluency, HaCaT cells were exposed to 5% PV-sera for another 24 h. The expression of Dsg3 was measured by WB using GAPDH as a loading control. The expression of Dsg3 declined over time.

Supplementary Figure 2. (A and B) HaCaT cells transfected with heat shock protein (HSP) 27 siRNA show markedly reduced HSP27 expression levels as compared with cells transfected with negative siRNA (scramble) and normal control group (NC). Levels of GAPDH are shown as a loading control. (C) Immunofluorescence staining of HaCaT cells transfected with HSP27 siRNA confirmed knockdown of HSP27 expression 72 h after transfection (scale bar = 200 μm).

Supplementary Figure 3. (A) HaCaT cells were cultured until 30–50% confluent and then transfected with heat shock protein (HSP)27 siRNA or negative siRNA. Then 48 h after transfection, the cells were mock-treated or treated with 5% pemphigus vulgaris (PV)-sera for another 24 h. HaCaT cells transfected with HSP27 siRNA showed markedly reduced HSP27 expression levels as compared with cells transfected with negative siRNA (scramble) and normal control group (NC) (A, lane 2). PV-sera induced Dsg3 depletion with silencing of HSP27 did not prevent loss of Dsg3 (A, lane 1). Levels of GAPDH are shown as a loading control (A, lane 3). (B) HSP27 siRNA silencing of HSP27 expression did not decrease loss of cell surface Dsg3 (red) after 24 h of treatment with 5% PV-sera; (scale bar = 200 μm).

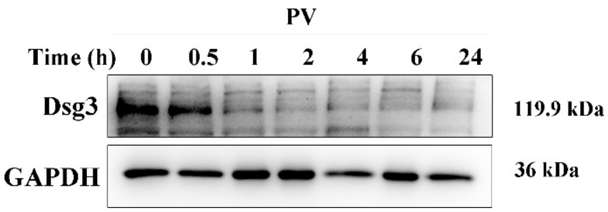

Supplementary Figure 1.

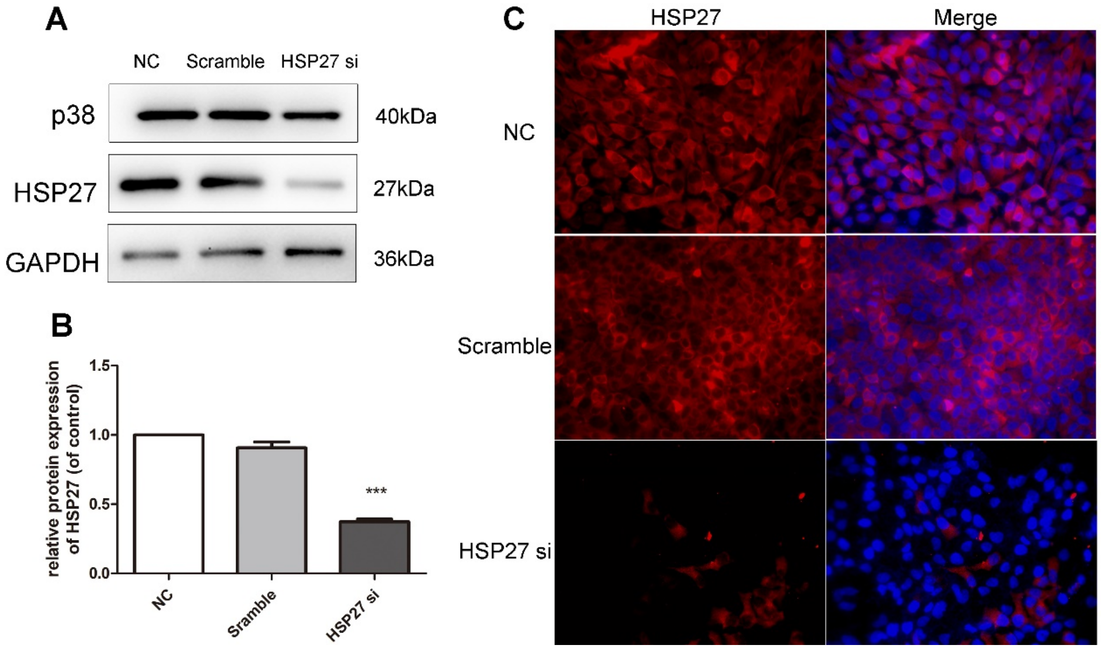

Supplementary Figure 2.

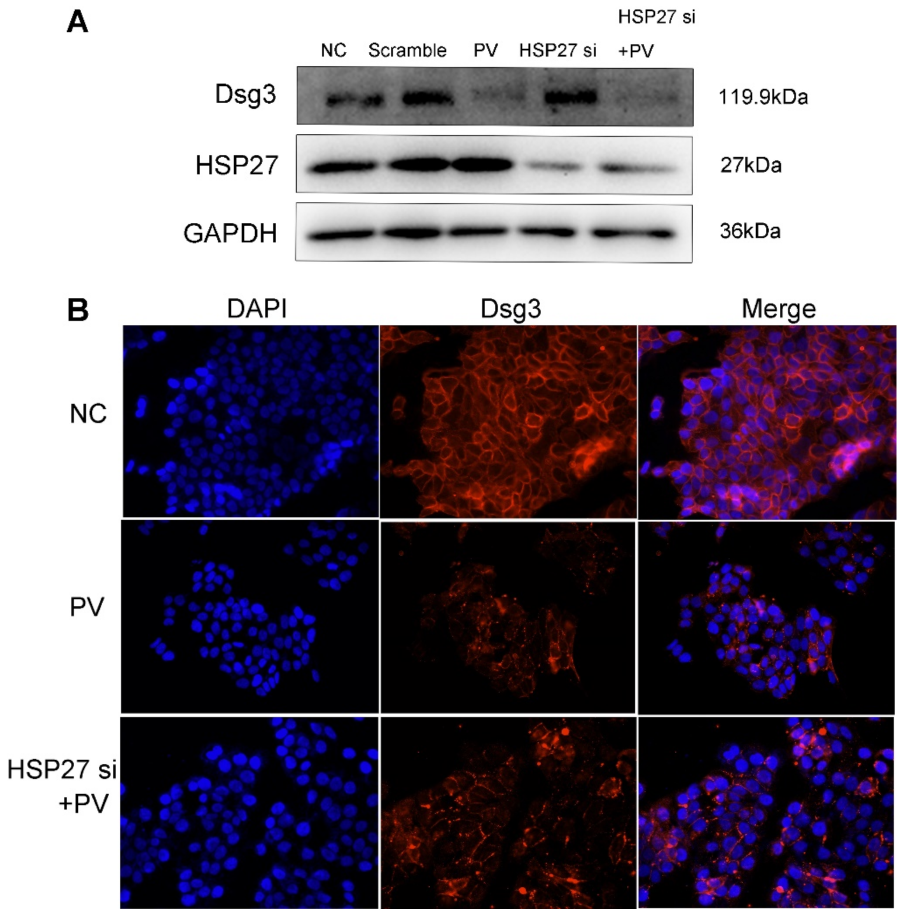

Supplementary Figure 3.
