# Supplementary material for: Tacrolimus reverses pemphigus vulgaris serum-induced depletion of desmoglein in HaCaT cells via inhibition of heat shock protein 27 phosphorylation
Source: BMC Immunol. 2023 Nov 8;24:43. doi: 10.1186/s12865-023-00582-z (PMC10634089; doi:10.1186/s12865-023-00582-z)
Supplement: Supplementary file 2 — Supplementary Material 2 [file 12865_2023_582_MOESM2_ESM.pdf]

Figure1

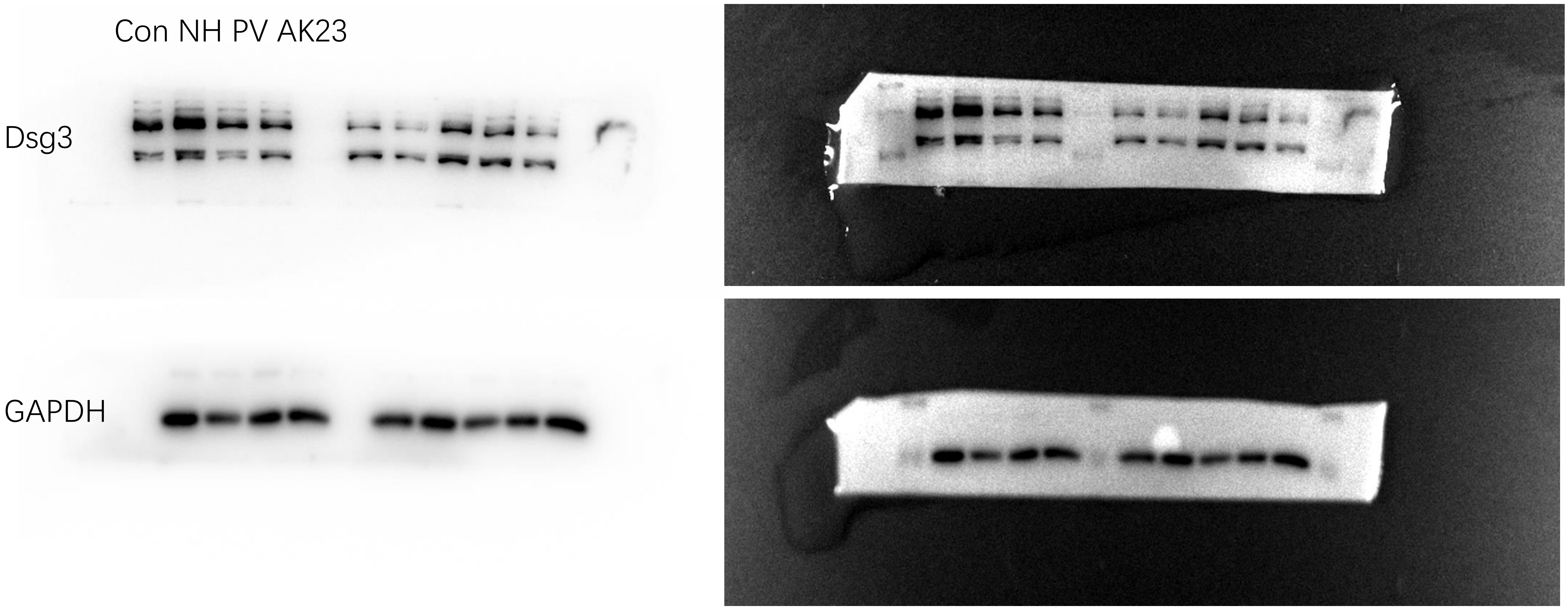

Figure 2A

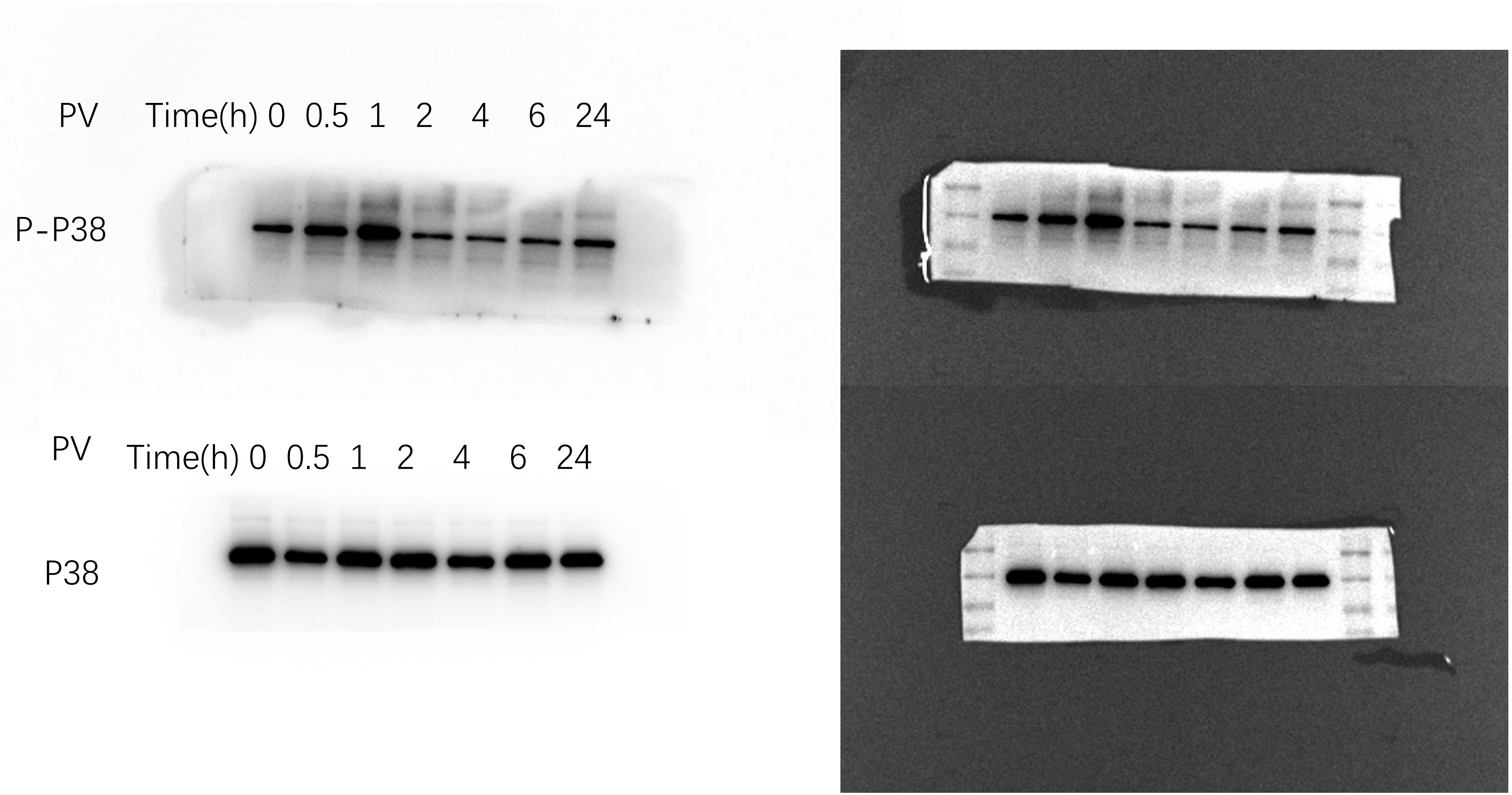

Figure2A

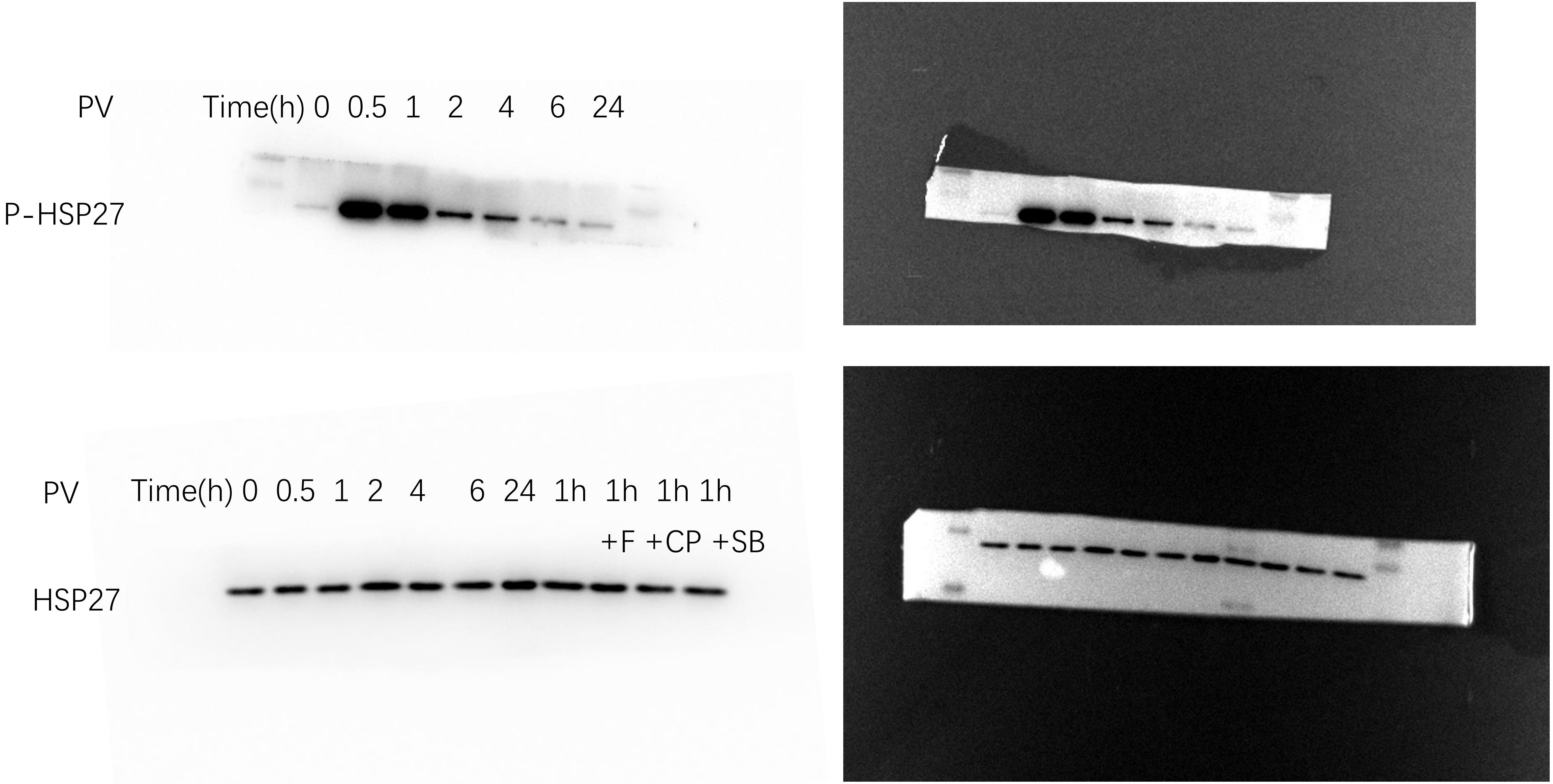
PV
Time(h) 0 0.5 1 2 4 6 24
P-HSP27
PV
Time(h) 0 0.5 1 2 4 6 24 1h 1h 1h 1h
+F +CP +SB
HSP27

Figure2A

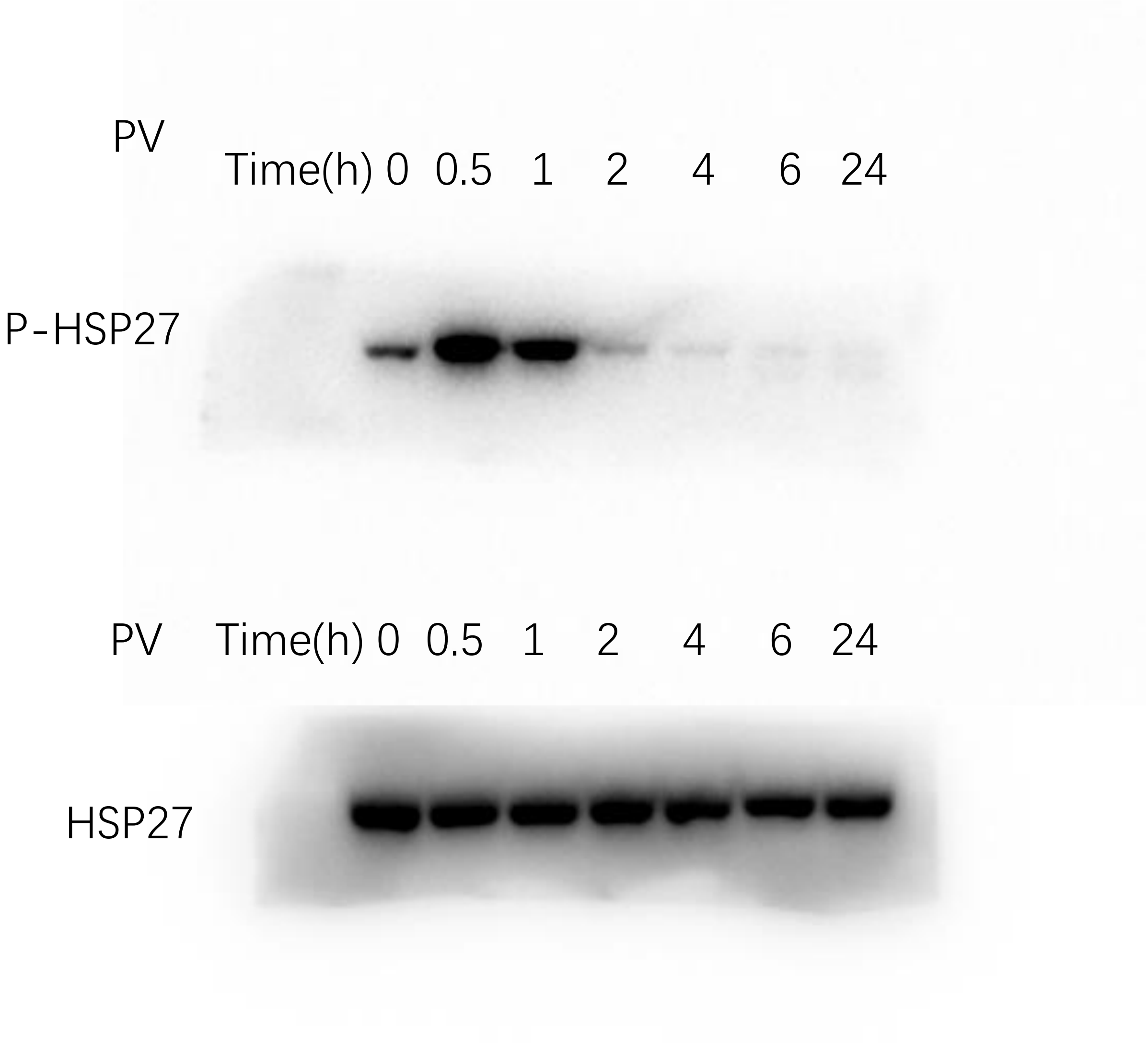

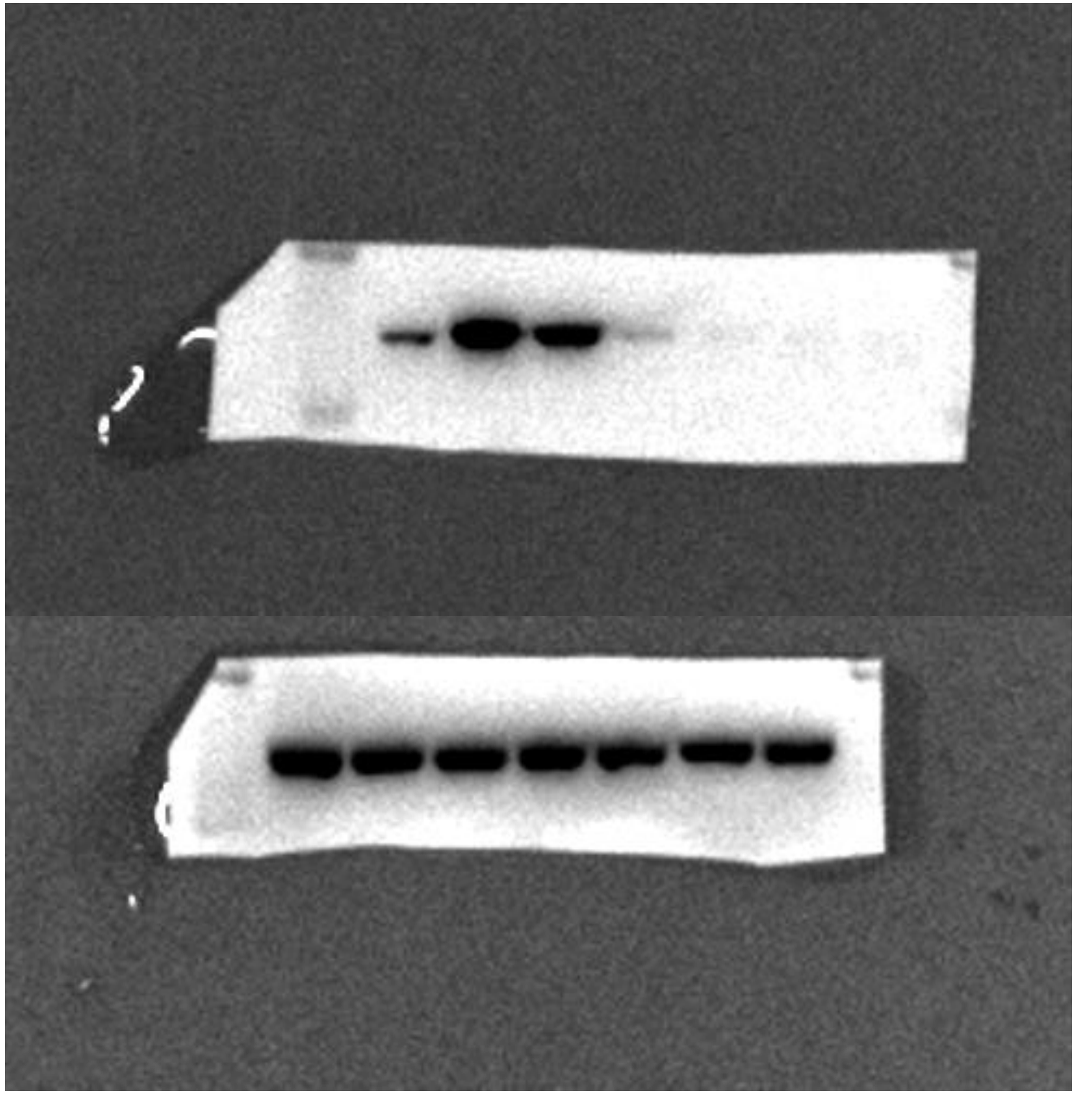

Figure2B

AK23 Time(h) 0 0.5 1 2 4 6 24

P-P38

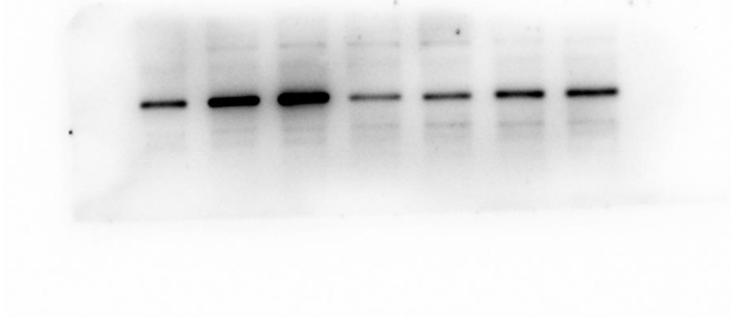

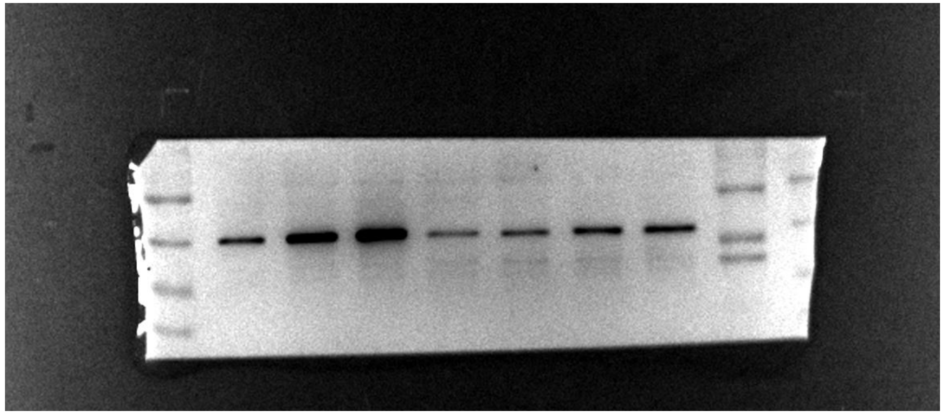

AK23 Time(h) 0 0.51 2 4 6 24

P38

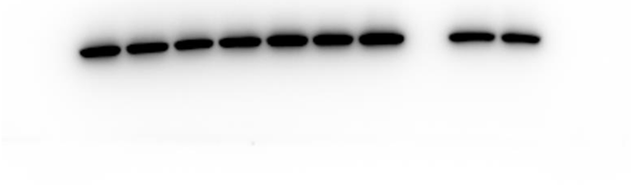

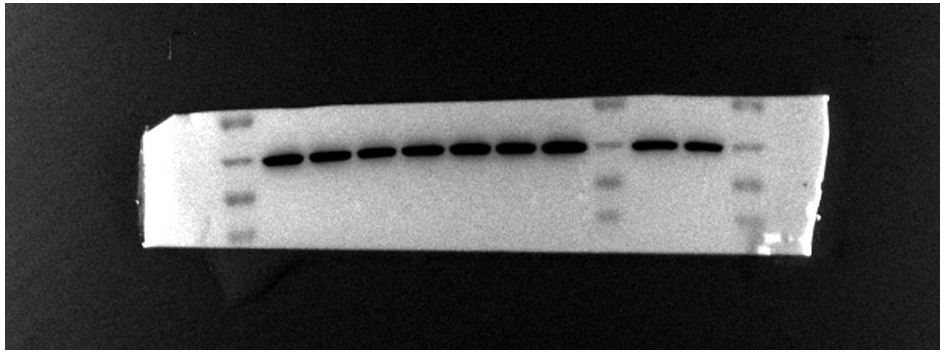

Figure2B

AK23 Time(h) 0 0.5 1 2 4 6 24

P-HSP27

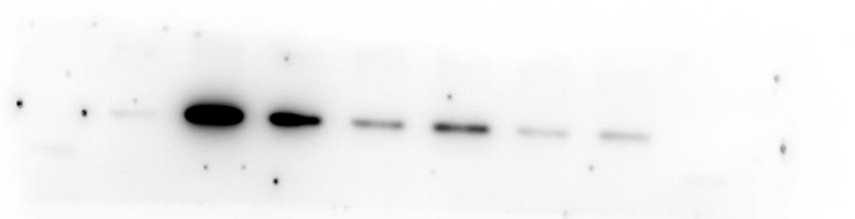

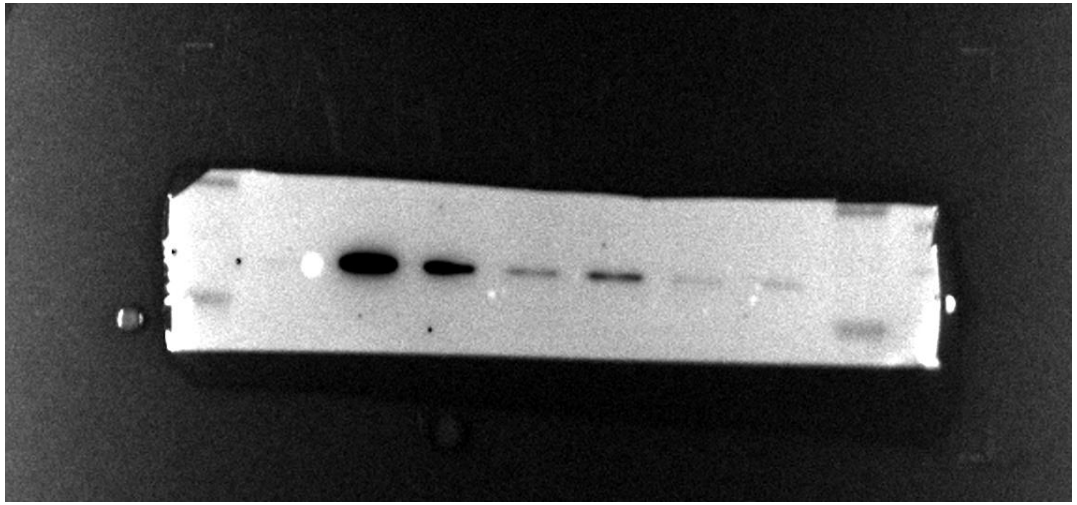

AK23 Time(h) 0 0.5 1 2 4 6 24 1h 1h 1h

+F +CP+SB

P-HSP27

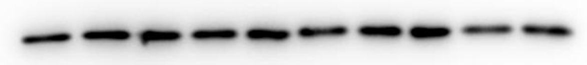

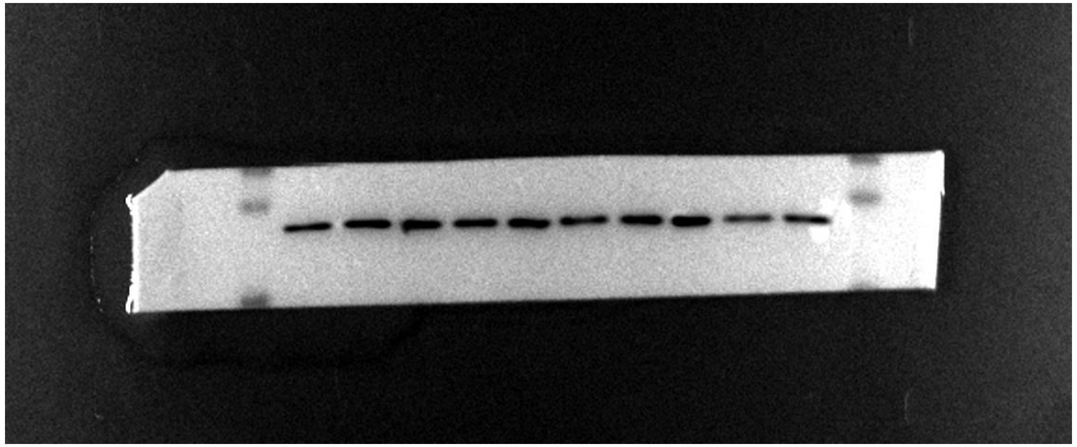

Figure3

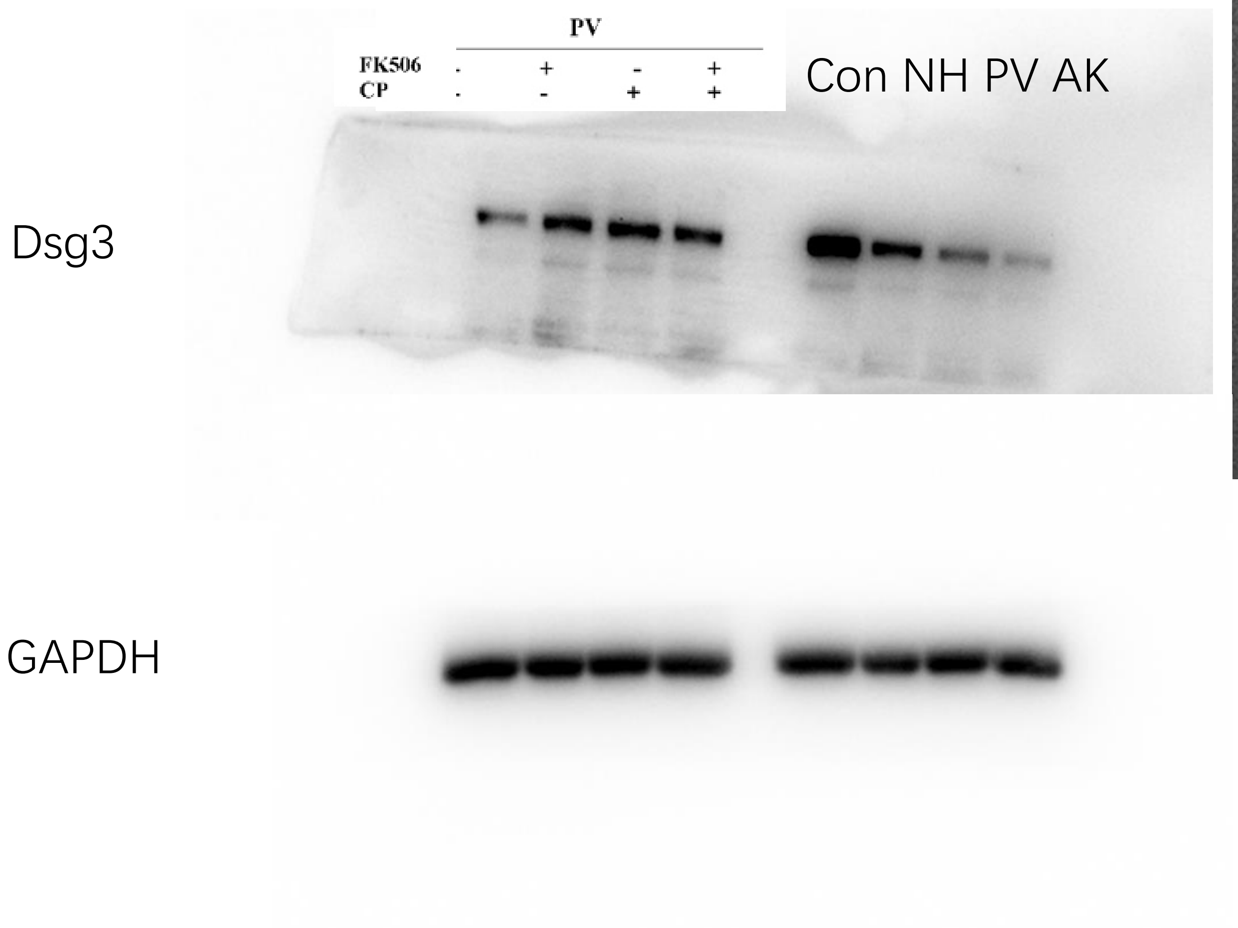

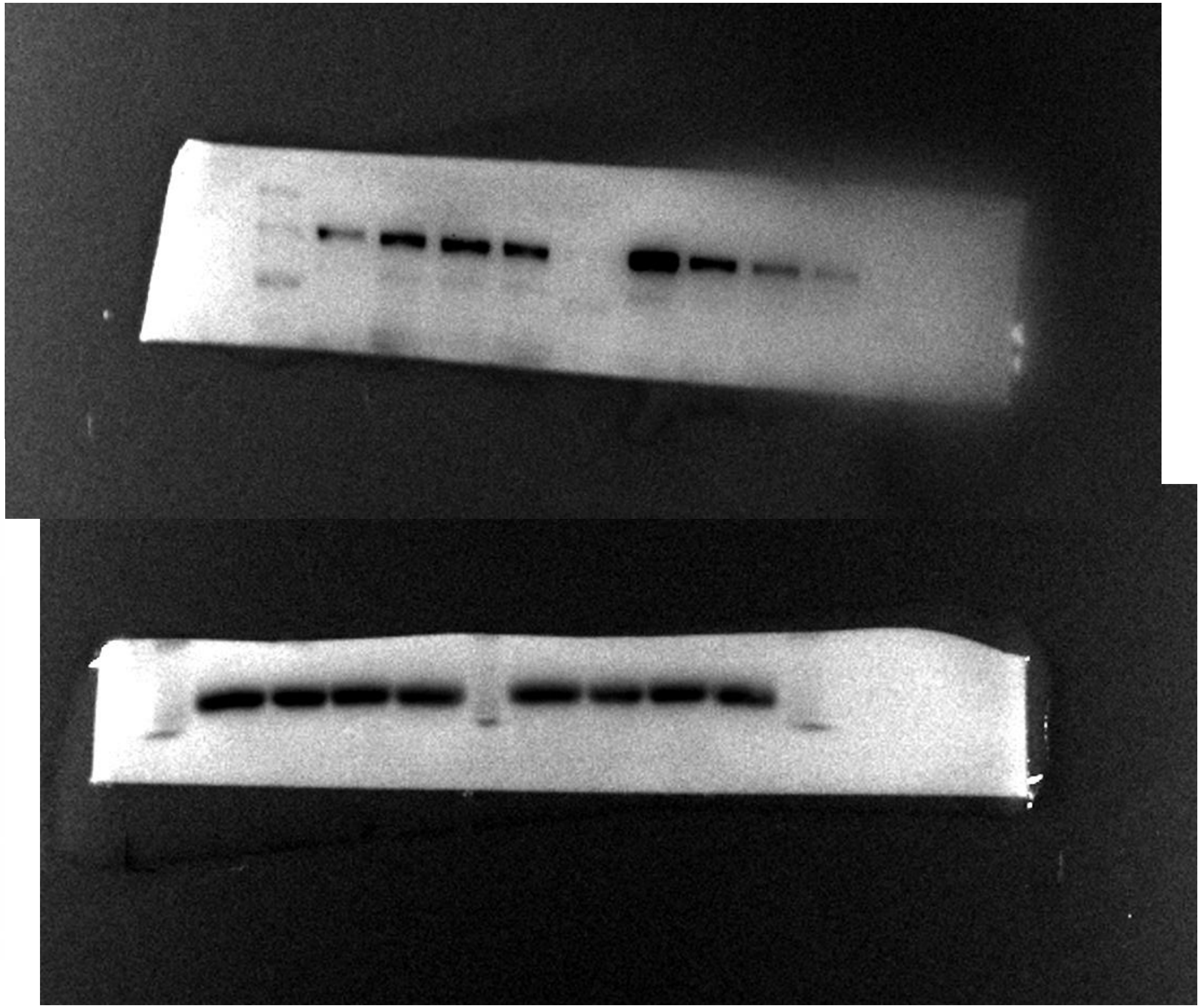

Figure3

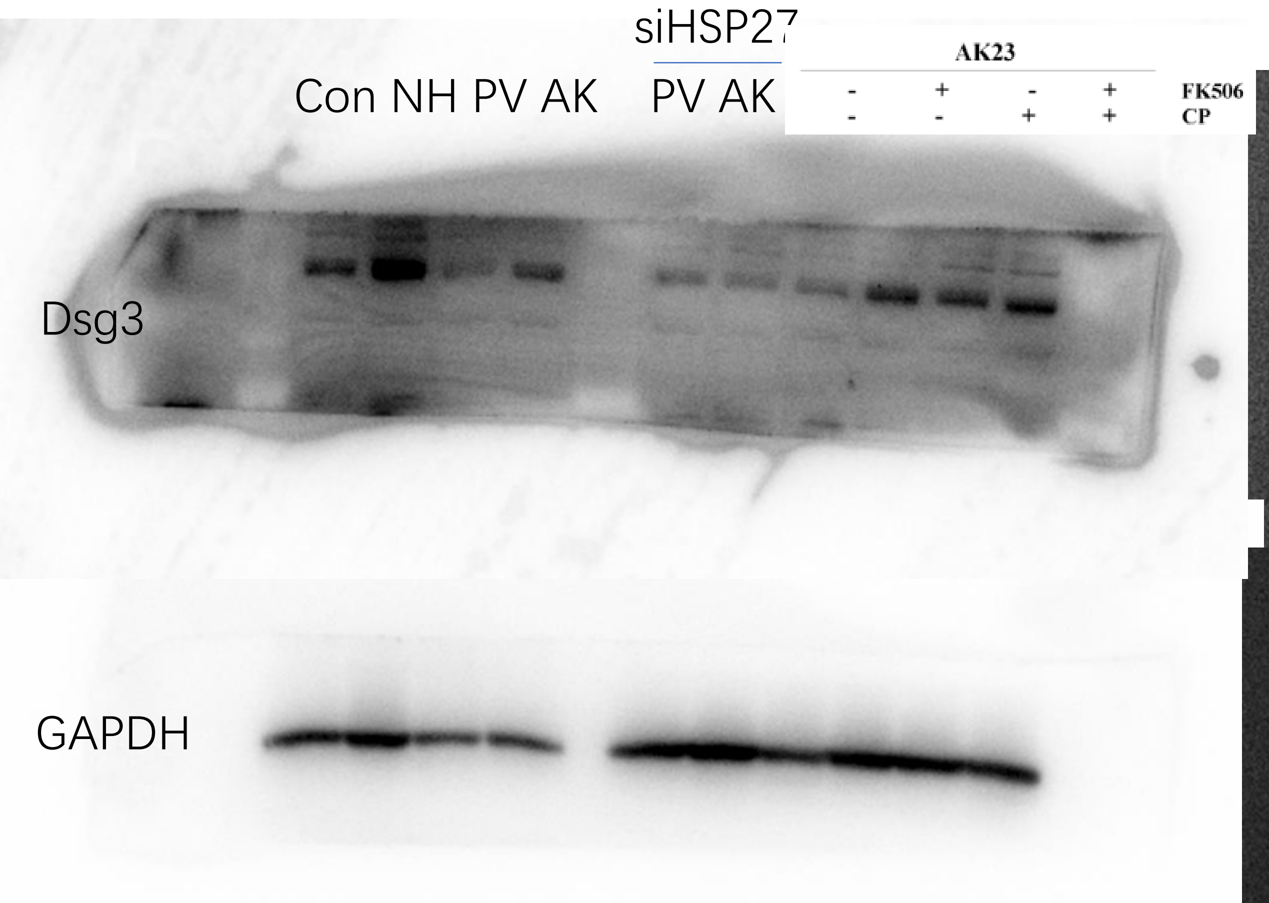

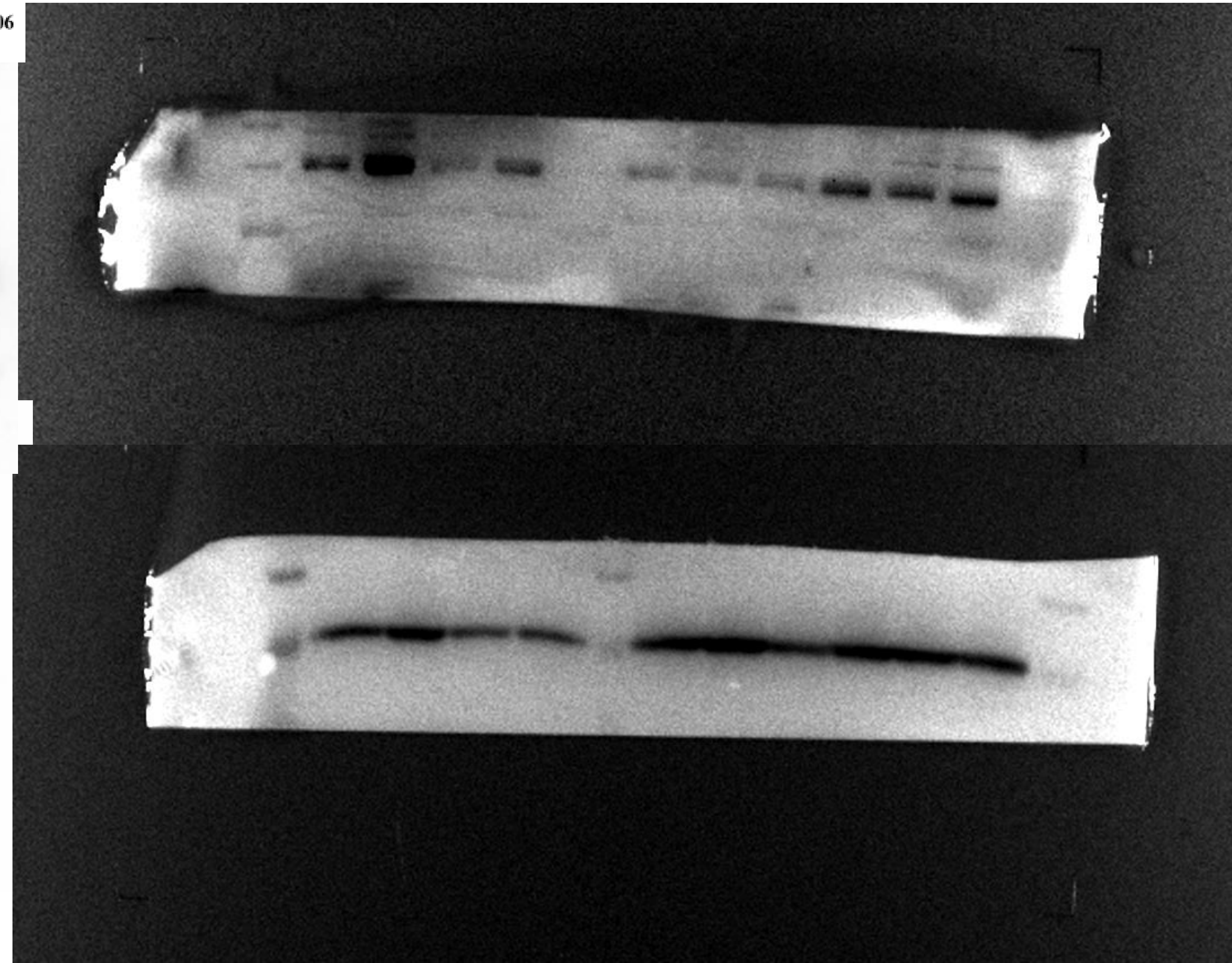

Figure4

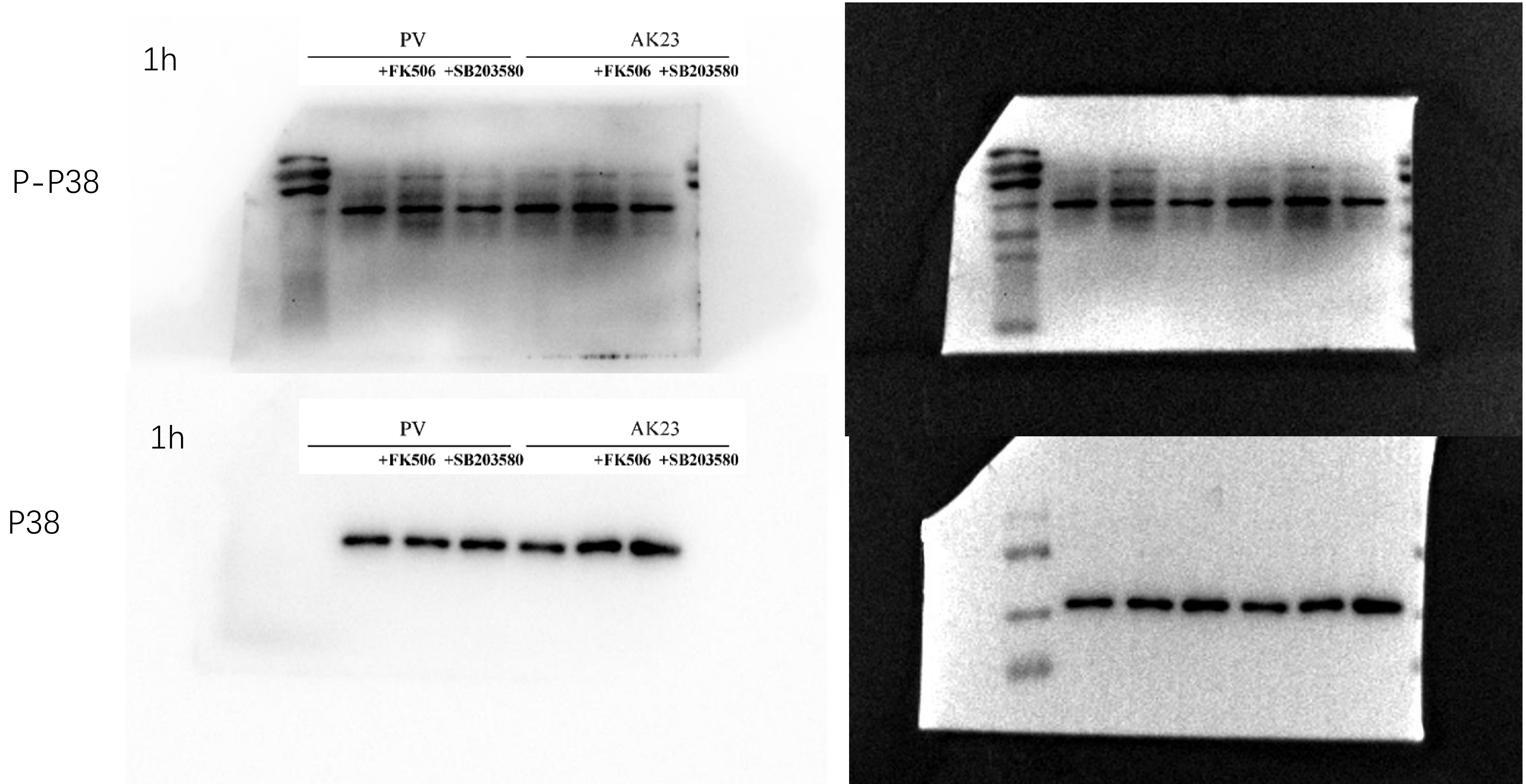
1h
PV
AK23
+FK506 +SB203580
+FK506 +SB203580
P-P38
1h
PV
AK23
+FK506 +SB203580
+FK506 +SB203580
P38

Figure4

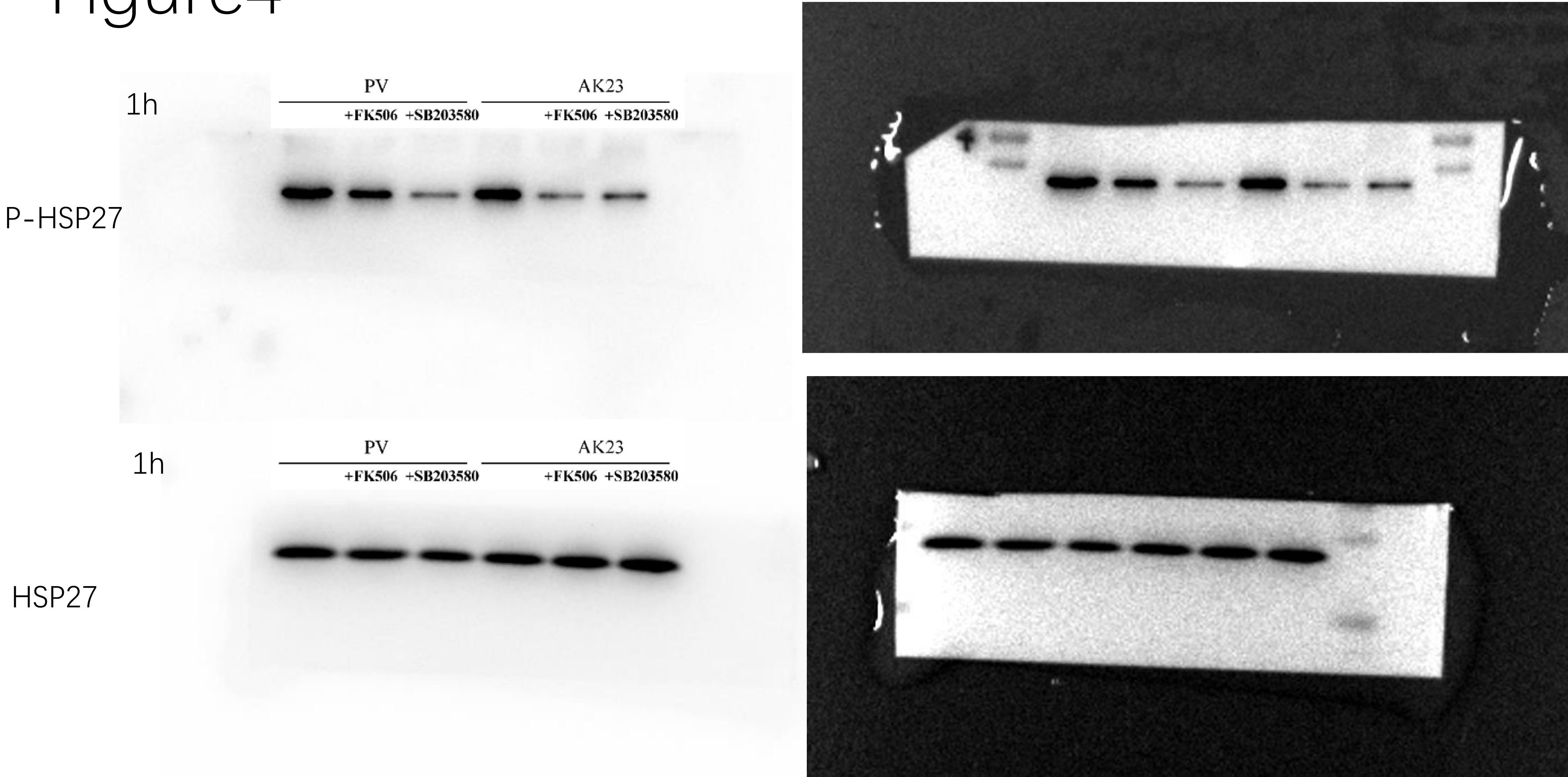
1h
PV
AK23
+FK506 +SB203580
+FK506 +SB203580
P-HSP27
1h
PV
AK23
+FK506 +SB203580
+FK506 +SB203580
HSP27
